# Psychological Distress among Hospitalized COVID-19 Patients in Denmark during the First 12 Months of the Pandemic

**DOI:** 10.3390/ijerph191610097

**Published:** 2022-08-15

**Authors:** Ellen Moseholm, Julie Midtgaard, Signe Bollerup, Ása D. Apol, Oskar B. Olesen, Sofie Jespersen, Nina Weis

**Affiliations:** 1 Department of Infectious Diseases, Copenhagen University Hospital, Hvidovre, Kettegaard Alle 30, 2650 Hvidovre, Denmark; 2 Department of Public Health, Faculty of Health and Medical Sciences, University of Copenhagen, Blegdamsvej 3B, 2200 Copenhagen N, Denmark; 3 Centre for Applied Research in Mental Health Care (CARMEN), Mental Health Centre Glostrup, University of Copenhagen, Nordstjernevej 41, 2600 Glostrup, Denmark; 4 Department of Clinical Medicine, Faculty of Health and Medical Sciences, University of Copenhagen, Blegdamsvej 3B, 2200 Copenhagen N, Denmark

**Keywords:** COVID-19, hospitalization, psychological distress, anxiety, depression, HRQoL

## Abstract

This study aimed to investigate psychological distress among patients hospitalized with a COVID-19 diagnosis in Denmark during the first 12 months of the pandemic and to assess changes in psychological distress in the three months following discharge. A single-center prospective observational survey study among patients hospitalized with a COVID-19 diagnosis between May 2020 and May 2021 was conducted. Participants completed a survey at three time points: at admission, and 1 and 3 months after discharge. Psychological distress was assessed by validated scales measuring symptoms related to depression, anxiety, stress, insomnia, post-traumatic stress disorder (PTSD), and health-related quality of life (HRQoL). In total, 95 patients were included. At admission, the proportion of patients with symptoms of depression was 43%, symptoms of anxiety 32%, moderate/high level of stress 39%, insomnia 52%, and probable/positive PTSD 19%. The burden of symptoms related to depression and anxiety decreased significantly over time, while there was no significant change over time in stress, insomnia, or PTSD. Suboptimal levels of physical and mental HRQoL were detected at admission but improved over time. Patients hospitalized due to COVID-19 during the first year of the pandemic experienced considerable levels of psychological distress at admission, with some improvements within 3 months after discharge.

## 1. Introduction

The first cases of coronavirus disease 19 (COVID-19), caused by the novel severe acute respiratory syndrome coronavirus 2 (SARS-CoV-2), were reported in December 2019. Since then, more than 430 million cases have been reported, and almost 6 million people have died from COVID-19 worldwide [1]. As the pandemic has spread, there has been a growing recognition of the psychological implications of COVID-19, both in the general population and in hospitalized patients [2,3,4]. Hospitalized COVID-19 patients may be particularly susceptible to adverse mental health conditions [5]. Several putative mechanisms by which COVID-19 may induce psychological symptoms have been proposed. First, it is well-known that being hospitalized for a serious illness can negatively affect mental health [3] and that this may be intensified when hospitalized with COVID-19 due to the exceptional circumstances within and outside the hospital during the pandemic [6]. Second, patients hospitalized with COVID-19 are isolated to avoid the virus spreading to other patients or healthcare staff. COVID-19 patients are thus limited in their access to social support, and their only in-person contact is with healthcare staff in full personal protective equipment. Studies have highlighted that isolation itself may have psychological implications [7,8,9]. Especially long periods of isolation seem to impact psychological well-being [7,9]. Finally, the host immune response to SARS-CoV-2 infection and the persistent psychological stress before and during infection, as well as adverse effects of treatment, such as insomnia caused by corticosteroids, have also been suggested as possible mechanisms [2].

The need to focus on the psychological impact of the COVID-19 pandemic alongside the physical symptoms of all those affected was highlighted by researchers and policymakers in the early phase of the pandemic [2,10]. Research conducted during the severe acute respiratory syndrome (SARS) and the Middle East Respiratory Syndrome (MERS) outbreaks has reported persistent psychological symptoms following hospitalization and isolation [11,12]. Thus, psychological symptoms due to serious coronavirus infections may have long-term health effects on patients [13], and focusing on mental health is important in the rehabilitation process of COVID-19 patients [14].

The current project was originally designed as an observational survey study with an embedded randomized controlled trial (RCT). The RCT aimed to test the efficacy of a minimal psychoeducational intervention versus standard of care at discharge for reducing symptoms of anxiety in hospitalized patients during the COVID-19 outbreak. However, with the emergence of different SARS-CoV-2 variants and more treatment options, the nature of the pandemic changed during the inclusion period, making it difficult to include the required number of participants in the RCT study. Hence, this manuscript presents the results of the observational survey study only. The purpose was to investigate psychological distress among patients hospitalized with COVID-19 diagnosis during the first 12 months of the pandemic and to assess changes in psychological distress in the three months following discharge. A secondary aim was to explore potential factors associated with the severity of the symptoms of depression and anxiety and health-related quality of life.

## 2. Materials and Methods

### 2.1. Study Design and Setting

This single-center prospective observational survey study using standardized and validated patient-reported outcome measures (PROMs) was conducted at Copenhagen University Hospital, Hvidovre, one of the major hospitals treating COVID-19 patients in Denmark. The study period was between 15 May 2020 and 15 May 2021, a period when the COVID-19 pandemic evolved in different ways over a relatively short time, including factors such as vaccination, treatment options, SARS-CoV-2 variants, and policy changes [3]. At the time when this study was initiated, the Danish population was subject to social isolation and mandatory quarantine and exposed to daily media reports on the raw number of those infected and deaths from COVID-19. In the early phase of the pandemic, 22% of all COVID-19 cases in Denmark were hospitalized [15], and the COVID-19 age-standardized mortality rate was, in 2020, 18.4/100,000 persons [16]. The study period coincided with the first and second COVID-19 peaks in Denmark.

### 2.2. Study Population

Patients were recruited from the Department of Infectious Disease and the COVID-19 isolation wards at the participating site. Eligible patients were consecutively identified at the time of admission. The inclusion criteria were adults ≥18 years of age, hospitalized with a confirmed COVID-19 diagnosis, able to read and understand Danish, and able to provide written informed consent. A COVID-19 diagnosis was confirmed by reverse transcriptase-polymerase chain reaction from oropharyngeal swaps. Patients were excluded if they were assessed to be cognitively incapable by their treating physician, had pulmonary cancer, or were terminally ill.

### 2.3. Data Collection

Data was collected with standardized questionnaires at three time points: the baseline completed within 48 h after admission (T1) and one month (T2) and three months (T3) after discharge. Patients answered the questionnaires electronically in REDCap© or via phone interviews conducted by a study nurse or medical student using a structured interview approach. All persons involved in the data collection received training to ensure uniformity and consistency. Clinical data were collected from the medical records and entered into the REDCap© database.

The following internationally well-known and validated PROMs were used to assess the psychological distress outcomes:

Symptoms of anxiety and depression were assessed using the Danish version of the Hospital Anxiety and Depression Scale (HADS) [17]. The scale is divided into an anxiety subscale (HADS-A) and a depression subscale (HADS-D), both containing seven intermingled items. Each item is scored between 0 and 3, where 0 is asymptomatic and 3 is severe. The total score for each of the two subscales ranges between 0 and 21, with higher scores indicating more severe symptoms of anxiety or depression [18,19]. A cutoff score of ≥8 is used to identify possible cases of clinical symptoms of anxiety and depression, as this cutoff has been shown to provide the optimal balance between specificity and sensitivity (between 0.70 and 0.90 for both scales) [18,19,20]. A score between 8 and 10 indicates mild symptoms of anxiety and depression, while a score of ≥11 indicates moderate/severe symptoms of anxiety/depression [18,19,20,21]. HADS has been validated in a Danish context [22].

Perceived stress was measured using the Perceived Stress Scale-10 item (PSS-10), a self-administered scale that was developed to measure “the degree to which situations in one’s life are appraised as stressful” during the past month [23]. Responses are in a Likert-type five-point format that ranges from 0 (never) to 4 (very often). The range for the scores is 0–40, with higher scores reflecting greater perceptions of stress. The PSS-10 has been translated and validated in Danish [24].

Sleep disturbance was measured using the Insomnia Severity Index (ISI), a 7-item scale assessing the nature, severity, and impact of insomnia within the past month [25,26]. A five-point Likert scale is used to rate each item (e.g., 0 = no problem; 4 = very severe problem), yielding a total score ranging from 0 to 28. The total score is interpreted as follows: absence of insomnia (0–7), sub-threshold insomnia (8–14), moderate insomnia (15–21), and severe insomnia (22–28). Previous studies have reported adequate psychometric properties for both the English and Danish versions [26,27].

Symptoms of post-traumatic stress disorder (PTSD) were measured using the 16 PTSD items based on DSM-III-R in the fourth section of The Harvard Trauma Questionnaire (HTQ) [28]. Each item is rated ranging from “Not at all” (score = 1) to “Extremely” (score = 4). A total score is calculated by the sum of the scores divided by the number of items answered, where less than 2 indicates no PTSD symptoms, 2–2.4 probable PTSD symptoms, and higher than 2.4 positive PTSD symptoms. The Danish version of the HTQ is both valid and reliable [29].

HRQoL was measured by SF-12, a short version of the Medical Outcomes Study (MOS) 36-item Short-Form Health Survey SF-36. The scale is designed to provide an assessment of general health concepts that are not specific to the age, disease, or treatment groups. The scale consists of 12 items addressing eight health concepts. Most questions refer to the past four weeks. In accordance with the manual, two summary scores ranging from 0 to 100 are calculated for physical health (PCS-12) and mental health (MCS-12), where a higher score equals a better HRQoL [30]. A score of 50 or less on the PCS-12 has been recommended as a cut-off to determine the affected physical health, while a score of 42 or less on the MCS-12 may be indicative of affected mental health [31]. The scale has been translated and validated in a Danish context [32].

### 2.4. Sociodemographic and Clinical Variables

The following demographic and clinical data were collected at the baseline (T1) from the patients’ records: age; sex; marital status; smoking status; alcohol use; nursing home residency; body mass index (BMI); previous history of psychiatric illness; physical symptoms; and clinical parameters, including temperature; oxygen support demands (<5 L O_2_/min, 5–10 L O_2_/min, and >10–30 O_2_/min); and mechanical ventilation needs. Data on the duration of hospital stays was collected at discharge. Self-reported information on country of birth, education, and employment was collected in the survey completed by patients at admission (T1).

### 2.5. Statistical Analysis

Descriptive statistics of the demographic characteristics at the baseline (T1) were computed. The results of the different psychological distress outcomes (depression, anxiety, stress, insomnia, PTSD, and HRQoL) were summarised at each time point (T1, T2, and T3), and linear mixed effects models for repeated measures were used to assess changes in the mean scores of each outcome over time. These models account for correlations between repeated measurements of the same individual over time and include all available data. Within-subject residuals were modeled with an unstructured variance–covariance structure. All mixed-effects models were bootstrapped with 2000 repetitions to account for the nonparametric distribution of data.

Bootstrapped univariate and multivariate linear mixed effect models were also used to assess the potential factors associated with the severity of symptoms of anxiety and depression and HRQoL. All variables with a *p*-value < 0.10 in the univariate mixed regression model were included in the multivariate mixed effect model to determine the potential factors significantly associated with the outcome (i.e., symptoms of anxiety and depression and HRQoL). Unknown/other/missing categories were included in the analysis. The reported coefficient (95% confidence interval (CI)) implies the estimated mean difference. Analyses were performed using STATA 17 software, and all reported *p*-values are two-sided using a significance level of 0.05.

### 2.6. Ethical Considerations

The study was approved by the Danish Data Protection Agency (P-2020-349). According to Danish Law, approval from the National Committee on Health Research Ethics was not required, as no biomedical intervention was performed. All participating patients provided written informed consent before any data collection.

## 3. Results

Overall, 433 patients were admitted with a COVID-19 diagnosis at the participating site during the study period. The reasons for ineligibility included: 127 (39%) could not speak or read Danish sufficiently, 50 (15%) were cognitively affected, 29 (9%) were severely ill, 42 (13%) were not able to complete the survey within 48 h of hospital admission, and 7 (2%) were discharged within 24 h. Thus, 178 patients were eligible for inclusion. In total, 107 (60%) of these patients agreed to complete the survey. Of these, 95 (53%) patients completed the baseline survey at admission (T1) and were thus included, 51 (54%) completed the survey at T2, and 45 (47%) completed the survey at T3. Additionally, 71 patients (40%) did not want to participate, most often because they were too fatigued or sick to complete the survey. Participating patients were more likely to be male compared to eligible, nonparticipating patients (71% versus 57%, *p* = 0.04), while there was no significant difference in age (median age: 61 versus 64 years, *p* = 0.36) or proportion born in Denmark (75% versus 64%, *p* = 0.17).

### 3.1. Study Participants

The characteristics of the participating patients are presented in Table 1. Most participating patients were married or living with a partner (76%), and more than half had a higher education (55%) and were currently employed (56%). More than half were former smokers (57%), while only 4% currently smoked. Overall, 54 (57%) participants had a Charlson’s Comorbidity Score of ≥1, and 26 (28%) had previously received treatment for mental illness; the most prevalent were treatments for depression (*n* = 13) and stress (*n* = 5). During admission, four (4%) patients developed the need for mechanical intervention treatment in the Intensive Care Unit (ICU), and nine patients died. The last patient was discharged on 16 May 2021, and the follow-up period continued until three months after this date (16 August 2021).

### 3.2. Psychological Distress Outcomes

**Figure 1:**
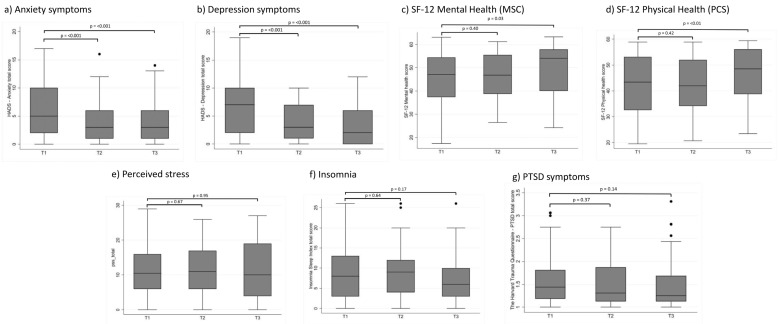
Psychological distress outcomes among hospitalized COVID-19 patients at admission (T1) and one month (T2) and three months (T3) after discharge.

Descriptive statistics of the psychological distress outcomes are presented in Table 2 and Figure 1. At T1, 43% (41/95) had possible symptoms of depression when using the recommended cutoff ≥ 8, of which 23% (*n* = 23) had mild symptoms of depression (HADS-D score 8–10), while 20% (*n* = 19) had moderate/severe symptoms of depression (score ≥ 11). There was a decrease over time, with 18% (8/45) reporting possible symptoms of depression at T3. The mean HADS-D score at T1 was 6.54 (95% CI: 5.60–7.47). There was a significant decrease in the HADS-D score over time (mean difference between T1 and T3 of −3.12 (95% CI: −4.25 to −1.98), *p* = <0.001).

The proportion of patients with possible symptoms of anxiety at T1 was 32% (30/95), of which 12% (*n* = 11) had mild symptoms of anxiety, while 20% (*n* = 19) had moderate/severe symptoms of anxiety. The proportion of participants with possible symptoms of anxiety decreased to 16% (8/51) at T2 and to 18% (8/45) at T3. The mean HADS-A score at admission was 5.87 (95% CI: 4.97–6.78). There was a significant decrease in the HADS-A score over time (mean difference between T1 and T3: −2.26 (95% CI: −3.33 to −1.19), *p* = <0.001).

The proportion of patients reporting moderate or high levels of perceived stress at T1 was 39% (37/94), 33% (16/49) at T2, and 38% (17/45) at T3. The mean perceived stress score at T1 was 11.11 (95% CI 9.70–12.51), and there was no significant change in the mean score over time.

The mean score on the insomnia severity index at admission was 8.58 (95% CI: 7.25–9.90). Half of the patients reported having some sleep problems at T1; however, only 14% (14/95) reported having moderate or severe clinical insomnia. There was no significant change in the insomnia scores over time (mean difference between T1 and T3: −1.24 (−3.02 to 0.53), *p* = 0.17). Sixty-two percent (28/45) of patients reported no sleep problems 3 months after admission, while 11% (5/45) reported moderate or severe clinical insomnia.

The mean PTSD score at T1 was 1.55 (95% CI: 1.45–1.65) and 6% (6/95) reported positive PTSD symptoms, while 13% (12/95) reported probable PTSD symptoms. There was no significant change in PTSD scores over time (mean difference between T1 and T3: −0.10 (−0.24 to 0.03), *p* = 0.14). The proportion of patients with positive PTSD scores at T3 was 9% (4/45), while the proportion of patients with probable PTSD symptoms was 7% (3/45).

The mean score on the SF-12 mental health component at T1 was 46.35 (95% CI: 44.15–48.55). There was a significant increase in the mental health scores over time (mean difference between T1 and T3: 3.47 (95% CI: 0.42 to6.52), *p* = 0.03), and the proportion of patients with affected mental health decreased from 69% (66/95) at T1 to 58% (26/45) at T3.

The mean score on the SF-12 physical component was 42.39 (95% CI: 40.15–44.63). There was a significant increase in the physical health scores over time (mean difference between T1 and T3: 4.24 (95% CI: 1.28 to7.19), *p* = <0.01), and the proportion of patients with affected physical health decreased from 34% (32/95) at T1 to 31% (14/45) at T3.

### 3.3. Potential Factors Associated with Symptoms of Anxiety and Depression and HRQoL

Table 3 presents the results from the analysis on the potential factors associated with the severity of the symptoms of anxiety and depression. The time since discharge, age, country of birth outside Denmark, education, and prior psychiatric treatment were associated with the severity of the anxiety symptoms in the univariate analysis and were included in the multivariate mixed effects analysis. The time since discharge remained significantly associated with lower HADS-A scores in the adjusted analysis (mean difference at 3 months (β = −2.15 (95% CI: −3.23 to −1.08), *p* = <0.001), while being born abroad was associated with a higher HADS-A score (β = 2.18 (95% CI: 0.36 to3.40), *p* = 0.02).

The time since discharge, country of birth outside Denmark, educational level, and comorbidity were associated with the severity of the depressive symptoms in the univariate analysis and were included in the multivariate mixed effects regression analysis. The time since discharge remained significantly associated with lower HADS-D scores in the adjusted analysis (mean difference at three months (β = −3.02 (95% CI: −4.17 to −1.87), *p* = <0.001). Being born abroad and a Charlson’s Comorbidity Score = 1 were associated with a higher HADS-D score (β = 2.27 (95% CI: 0.48–4.04), *p* = 0.01 and β = 1.74 (95% CI: 0.37 to 3.11), *p* = 0.01, respectively).

Table 4 presents the results of the analysis of the potential factors associated with the mental health and physical health components of the SF-12 scale. The time since discharge, comorbidity, severity of anxiety symptoms, severity of depressive symptoms, severity of perceived stress, severity of insomnia, and severity of PTSD symptoms were associated with affected mental health in the univariate analysis and were included in the multivariate mixed effects regression analysis. A Charlson’s Comorbidity Score = 2 was negatively associated with mental health in the adjusted analysis (β = −4.77 (95% CI: −7.14 to −2.39), *p* = <0.001). The severity of the depressive symptoms and severity of perceived stress were also negatively associated with mental health (β = −0.77 (95% CI: −1.16 to −0.37), *p* = <0.001 and β = −0.43 (95% CI: −0.68 to −0.19), *p* = <0.01, respectively).

The time since discharge, sex, comorbidity, severity of anxiety symptoms, depressive symptoms, perceived stress, insomnia, and PTSD symptoms were associated with affected physical health in the univariate analysis and were included in the multivariate mixed effects regression analysis. Female sex was negatively associated with physical health in the adjusted analysis (β = −3.50 (95% CI: −6.28 to −0.72), *p* = 0.01). A Charlson’s Comorbidity Score = 1 or 2 was also negatively associated with physical health in the adjusted analysis (β = −2.98 (95% CI: −5.68 to −0.29), *p* = 0.03 and β = −4.69 (95% CI: −7.94 to −1.45), *p* = <0.01, respectively), as was the severity of the depressive symptoms (β = −0.58 (95% CI: −0.97 to −0.18), *p* = <0.01), perceived stress (β = −0.50 (95% CI: −0.79 to −0.22), *p* = <0.01), and insomnia (β = −0.32 (95% CI: −0.57 to −0.07), *p* = 0.01).

## 4. Discussion

In this observational study executed in the first year of the pandemic, we found that, at the time of admission, more than one-third of patients hospitalized for COVID-19 reported clinically relevant symptoms of psychological distress. Symptoms of depression and anxiety and perceived stress seemed to be especially prevalent. The burden of symptoms related to depression and anxiety decreased significantly over time, while there was no significant change in perceived stress, insomnia, or symptoms of PTSD over time. HRQoL, measured by the SF-12 mental and physical components, was also impacted at the time of admission but improved over time. These findings highlight the burden of psychological distress over time in hospitalized COVID-19 patients, which has not been described previously in a Danish context.

### 4.1. Psychological Distress during and after Hospital Admission

The proportion of participants with probable symptoms of depression was 43% and 32% of anxiety at the time of admission. This is in line with other studies conducted during the same time period [33,34,35], including a systematic review reporting that 9–66% of patients hospitalized for COVID-19 may experience depressive symptoms, while 30–39% may experience anxiety symptoms [3]. The time of data collection in the studies included in this review varied considerably from collection during hospitalization and up to three months following discharge, which might explain the variability of their results. Similar to our study, Sahan et al. [33] reported a prevalence of depression of 42% and anxiety of 35% among hospitalized COVID-19 patients in Turkey, with a decrease in both scores over time. We also found a significant decrease in depression and anxiety scores over time, and our findings are very much in line with other studies focusing on psychological distress following discharge [6,36].

Close to 40% of the participants in our study reported moderate or high perceived stress levels, with no change over time. The COVID-19 pandemic has profoundly disrupted the daily routines of individuals with quarantine measures, travel bans, and uncertainties due to the unpredictable future of the pandemic [37]. This may, in turn, be associated with increased perceptions of stress in people requiring inpatient care, especially in the early phases of the pandemic, during which our study was conducted. A Chinese qualitative study conducted among 16 COVID-19 patients after hospital discharge showed that patients may experience concerns about the recurrence of disease, physical sequelae, stigma, death of family members due to COVID-19, and financial stress [38]. As daily stress predicts long-term health and well-being [39,40], it is important to understand perceived stress and the associated protective factors among hospitalized COVID-19 patients. More research is needed to explore this.

More than half of the participants in this study experienced some level of sleep disturbances, with no significant change over time. Wang et al. [41] found, in their study among 484 hospitalized COVID-19 patients in Wuhan, China, a 43% prevalence of insomnia disorder. These findings were supported in a large systematic review and meta-analysis evaluating the extent of sleep disturbances during the COVID-19 pandemic, where the estimated prevalence of sleep problems was 52.4% (95% CI: 41.7–62.9%] among patients infected with COVID-19 (hospitalization status not specified) [42]. The estimated global prevalence of sleep disturbances was 40.5%, highlighting that sleep disturbances were common during the COVID-19 pandemic [42]. This may explain our finding that no change was found in insomnia over time.

Follow-up studies conducted in the early phases of the pandemic among COVID-19 patients 1–3 months after their discharge have reported a prevalence of PTSD ranging from 12 to 22% [14,43]. This is higher than the prevalence found in our study. Possible explanations for this difference could be: timing in relation to the evolution of the pandemic, differences in the instrument used to assess PTSD, and different clinical definitions of PTSD. Similar to our findings, a Norwegian study found a 9.5% prevalence of PTSD symptoms among COVID-19 patients three months after discharge [44]. Although nonsignificant, a small increase in the proportion of positive PTSD symptoms was observed over time. PTSD symptoms may develop over time beyond the follow-up time of our study [12,45], and thus, longer follow-up periods are needed to assess the occurrence of PTSD in discharged COVID-19 patients.

Improvements in HRQoL measured by the mental and physical components of the SF-12 instrument were observed over time. However, at three months after discharge, the proportion of patients with especially affected mental health remained high. These findings were supported by Vlake et al. [6]. A recent review also found that, regardless of the time since discharge, HRQoL was significantly impacted among COVID-19 patients [46]. COVID-19 infections may cause long-term problems such as fatigue, coughing, and shortness of breath [47], all symptoms that could impact HRQoL. While follow-up clinics for COVID-19 patients have been implemented in the Danish healthcare system [48], these focus on patients with severe physical long-term effects following a COVID-19 infection. Thus, strategies for improving all aspects of HRQoL in discharged COVID-19 patients appear warranted.

### 4.2. Potential Factors Associated with Symptoms of Depression and Anxiety and HRQoL

The time since discharge and country of birth outside of Denmark were negatively associated with the severity of symptoms of anxiety and depression. This finding is supported by other studies [6,36]. Ethnic minorities have been shown to have disproportionately higher risks of being adversely affected by COVID-19, both in relation to diagnosis and severity of infection [49]. Physical and psychological health vulnerabilities are inextricably linked, and given the higher risks of mental illnesses and complex care needs among ethnic minorities, this group of patients may be particularly vulnerable to long-term psychological distress following a COVID-19 infection [50]. Unlike other studies [6,36], we did not find female sex to be associated with the severity of anxiety symptoms or depressive symptoms. This may be explained by the low number of women included in our study.

Comorbidity, the severity of depression, and perceived stress were negatively associated with both physical and mental HRQoL. These findings are supported by other studies [6,46]. Similar to Vlake et al. [6], we found that female sex was associated with physical HRQoL but not mental HRQoL. Insomnia was also negatively associated with physical HRQoL. Many of these factors can be addressed with preventive interventions focusing on lowering anxiety and stress levels, which, in turn, could positively impact HRQoL. Although validated interventions focusing specifically on psychological distress in hospitalized patients with COVID-19 are still limited and not yet of sufficient quality to inform the practice [51], psychoeducational interventions have been shown to significantly improve mental health-related issues in this population [52]. Moreover, the increased knowledge about the behavior of the virus and the now available treatment options may contribute to lower stress and anxiety levels among more recently hospitalized patients compared to patients hospitalized closer to the beginning of the pandemic [3]. Thus, informing patients hospitalized with COVID-19 about the treatment options and prognosis related to the current variants of the SARS-CoV-2 virus may help improve their mental health and HRQoL.

### 4.3. Possible Practical Implications for Healthcare Professionals

Healthcare professionals should be aware of and acknowledge the psychological implications that hospitalization due to COVID-19 may have on the patient. Recommendations for mental health professionals have been published [53,54], highlighting the basic principles for the incorporation of mental healthcare in the management of the COVID-19 pandemic. Ideally, an integrated continuum of care approach focusing on vulnerable patients should be implemented involving the mental health services and the somatic wards treating patients with COVID-19. Patients with an ethnic minority background and patients experiencing psychological distress at the time of admission may be particularly vulnerable to negative long-term psychological effects of COVID-19 [54]. The provision of staff guidelines for COVID-19 patients, e.g., protocols for screening for symptoms of anxiety, may help healthcare professionals identify and support patients who experience psychological distress during admission [55]. Ongoing and future studies will hopefully provide more robust knowledge about validated psychological support interventions that may be suitable for implementation during and following a hospitalization with COVID-19-related illness.

### 4.4. Strengths and Limitations

To our knowledge, this is the first study to assess the psychological distress among patients hospitalized due to COVID-19 in Denmark during the first 12 months of the pandemic. The small sample size is a limitation. Most of the participating patients had mild or moderate symptoms, which limited our ability to assess the association between the severity of the disease and symptoms of psychological distress. Moreover, the single-center design limited the external validity. However, the study was conducted at one of the major hospitals treating COVID-19 patients in Denmark, and national in-hospital precautionary measures to limit the spread of the virus, as well as treatment protocols, were timely implemented throughout the study period. Psychological distress was assessed using self-reported questionnaires. Although commonly used and widely validated, the usage of self-reports may result in an overestimation of psychologic distress. A formal assessment of psychiatric disorders requires consultation with a psychologist or psychiatrist.

## 5. Conclusions

This single-center observational study conducted among hospitalized COVID-19 patients in Denmark found a high proportion of psychological distress at the time of admission. Symptoms of depression and anxiety and perceived stress were especially prevalent. The burden of symptoms related to depression and anxiety decreased significantly over time, while there was no significant change in perceived stress, insomnia, or symptoms of PTSD over time. Physical and mental HRQoL were also impacted at the time of admission but improved over time. Long-term follow-up is needed to assess the impact of COVID-19 on psychological distress beyond three months after admission.

## Figures and Tables

**Table 1:** Patient characteristics at hospital admission (*n* = 95).

**Age**	
Median (IQR)	61 (49–72)
Range	24–90
**Sex, *n* (%)**	
Male	68 (72)
Female	27 (28)
**Born in Denmark**	
Yes	70 (75)
No	24 (25)
**Relationship status**	
Married/living with partner	72 (76)
Has a partner, but not living together	7 (7)
No partner	16 (17)
**Nursing home resident, *n* (%)**	<3 (1)
**Education**	
Compulsory (middle/high school)	40 (43)
Higher education (college/university)	51 (55)
Missing	<3 (2)
**Currently employed**	
Yes	53 (56)
No	40 (42)
Missing	<3 (2)
**BMI**	
Median (IQR)	29.4 (25.8–32.0)
Range	20.4–46.1
Missing	4
**Smoking**	
Never smoked	37 (39)
Former smoker	54 (57)
Current smoker	4 (4)
**Alcohol use**	
Greater than recommendations	12 (13)
Less than recommendations	83 (87)
**Charlson’s Comorbidity Index**	
0	41 (43)
1	32 (34)
≥2	22 (23)
**Ever treated for a psychological problem**	
Yes	26 (28)
No	65 (69)
Missing	3 (3)
**Temperature**	
Median (IQR)	37.4 (36.6–38.5)
Range	35.7–40.9
**Oxygen need**	
<5 L O_2_/min	62 (65)
5–10 L O_2_/min	19 (20)
11–29 L O_2_/min	13 (14)
>30 L O_2_/min	<3 (1)
**Hospital length of stay, days, median (IQR)**	6 (2–24)
**Admitted to ICU for mechanical intervention, *n* (%)**	4 (4)
**Died during hospital admission**	9 (9)

BMI = Body Mass Index; ICU = Intensive Care Unit.

**Table 2:** Psychological distress outcomes over time among 95 admitted COVID-19 patients during May 2020–May 2021.

		T1	T2	T3	Difference between T1 and T3		Difference between T1 and T3	
	Range				Mean (95% CI)	*p*-Value	Mean (95% CI)	*p*-Value
**Symptoms of depression (HADS-D)**	0–21	*n* = 95	*n* = 51	*n* = 45				
Mean score (95% CI)		6.54 (5.60–7.47)	3.71 (2.78–4.63)	3.29 (2.17–4.41)	−2.77 (−3.87 to −1.67)	**<0.001**	−3.12 (−4.25 to −1.98)	**<0.001**
No depression, (score < 8), *n* (%)		54 (57)	41 (84)	37 (82)				
Possible depression, (score ≥ 8), *n* (%)		41 (43)	8 (16)	8 (18)				
**Symptoms of anxiety (HADS-A)**	0–21	*n* = 95	*n* = 51	*n* = 45				
Mean score (95% CI)		5.87 (4.97–6.78)	3.90 (2.82–4.98)	3.93 (2.77–5.09)	−2.27 (−3.35 to −1.20)	**<0.001**	−2.26 (−3.33 to −1.19)	**<0.001**
No anxiety (score < 8), *n* (%)		65 (68)	43 (84)	37 (82)				
Possible anxiety, (score ≥ 8), *n* (%)		30 (32)	8 (16)	8 (18)				
**Perceived Stress (PSS)**	0–40	*n* = 94	*n* = 49	*n* = 45				
Total score, mean (95% CI)		11.11 (9.70–12.51)	11.47 (9.42–13.52)	10.93 (8.56–13.29)	0.41 (−1.56 to 2.38)	0.67	−0.09 (−2.60 to 2.43)	0.95
Low stress (score 0–13), *n* (%)		57 (61)	33 (67)	28 (62)				
Moderate stress (score 14–26), *n* (%)		35 (37)	16 (33)	16 (36)				
High stress (score 27–40), *n* (%)		2 (2)	0	1 (2)				
**Insomnia (ISI)**	0–28	*n* = 95	*n* = 49	*n* = 45				
Mean score (95% CI)		8.58 (7.25–9.90)	9.41 (7.59–11.22)	7.33 (5.51–9.16)	0.34 (−1.11 to 1.79)	0.64	−1.24 (−3.02 to 0.53)	0.17
No insomnia (score 0–7), *n* (%)		45 (48)	19 (39)	28 (62)				
Subthreshold insomnia (score 8–14), *n* (%)		36 (38)	20 (41)	12 (27)				
Clinical insomnia—moderate (score 15–21), *n* (%)		11 (11)	8 (16)	4 (9)				
Clinical insomnia—severe (score 22–28), *n* (%)		3 (3)	2 (4)	1 (2)				
**PTSD Symptoms (HTQ)**	1–4	*n* = 94	*n* = 49	*n* = 45				
Mean score (95% CI)		1.55 (1.45–1.65)	1.53 (1.38–1.68)	1.47 (1.31–1.63)	−0.05 (−0.15 to 0.06)	0.37	−0.10 (−0.24 to 0.03)	0.14
No PTSD symptoms, (score < 2), *n* (%)		76 (81)	40 (82)	38 (84)				
Probable PTSD Symptoms, (score 2–2.4), *n* (%)		12 (13)	4 (8)	3 (7)				
Positive PTSD Symptoms, (score > 2.4), *n* (%)		6 (6)	5 (10)	4 (9)				
**SF-12: Mental health score (MSC)**	0–100	*n* = 95	*n* = 51	*n* = 45				
Mean score (95% CI)		46.35 (44.15–48.55)	46.42 (43.60–49.23)	49.33 (46.05–52.62)	1.06 (−1.39 to 3.51)	0.40	3.47 (0.42 to6.52)	**0.03**
Affected mental health (score ≤ 42), *n* (%)		66 (69)	34 (67)	26 (58)				
**SF-12: Physical health score (PSC)**		*n* = 95	*n* = 51	*n* = 45				
Mean score (95% CI)		42.39 (40.15–44.63)	42.48 (39.42–45.54)	46.26 (43.04–49.47)	1.21 (−1.71 to 4.13)	0.42	4.24 (1.28 to7.19)	**<0.01**
Affected physical health (score ≤ 50), *n* (%)		32 (34)	16 (31)	14 (31)				

Linear mixed effects models for repeated measures were used to assess changes in the mean scores of the different psychological distress outcomes over time. Significant changes defined as *p*-values < 0.5 are highlighted in bold. T1 =< 48 h following hospital admission, T2 = one month after hospital discharge, and T3 = three months after hospital discharge.

**Table 3:** Potential factors associated with the severity of symptoms of anxiety and depression among 95 admitted COVID-19 patients during May 2020–May 2021.

	Severity of Anxiety Symptoms				Severity of Depressive Symptoms			
	Univariate		Multivariate		Univariate		Multivariate	
	Beta (95% CI)	*p*-Value	Beta (95% CI)	*p*-Value	Beta (95% CI)	*p*-Value	Beta (95% CI)	*p*-Value
**Time since discharge**								
1 month	−2.27 (−3.34 to −1.20)	**<0.001**	−2.11 (−3.18 to −1.04)	**<0.001**	−2.77 (−3.87 to −1.67)	**<0.001**	−2.62 (−3.74 to −1.49)	**<0.001**
3 months	−2.26 (−3.33 to −1.19)	**<0.001**	−2.15 (−3.23 to −1.08)	**<0.001**	−3.12 (−4.25 to −1.98)	**<0.001**	−3.02 (−4.17 to −1.87)	**<0.001**
**Age, years**	−0.06 (−0.09 to −0.03)	**<0.001**	−0.03 (−0.08 to 0.01)	0.17	−0.02 (−0.06 to 0.02)	0.38		
**Sex (female)**	1.17 (−0.88 to 2.46)	0.07	1.03 (−0.35 to 2.41)	0.15	1.21 (−0.18 to 2.60)	0.09	0.64 (−0.69 to 1.98)	0.35
**County of birth outside DK**	3.51 (2.10–4.92)	**<0.001**	2.18 (0.36 to3.40)	**0.02**	2.76 (1.27 to4.24)	**<0.001**	2.27 (0.48 to4.04)	**0.01**
**Education**								
Compulsory (middle/high school)	ref		ref		ref		Ref	
Higher education (College/University)	−1.43 (−2.61 to −0.24)	**0.02**	−0.60 (−1.88 to 0.72)	0.38	−1.77 (−2.95 to −0.59)	**<0.01**	1.05 (−0.98 to 3.07)	0.31
**Charlson’s Comorbidity**								
0	ref				ref			
1	0.34 (−1.11 to 1.80)	0.64			1.67 (0.24 to3.09)	**0.02**	1.74 (0.37 to3.11)	**0.01**
2	−0.62 (−1.95 to 0.71)	0.35			0.94 (−0.41 to 2.28)	0.17	1.24 (−0.36 to 2.83)	0.13
**Hospital stay, days**	0.01 (−0.02 to 0.03)	0.62			−0.01 (−0.08 to 0.07)	0.95		
**ICU admission/mechanical intervention**	−0.98 (−2.40 to 0.45)	0.18			3.17 (−0.57 to 6.93)	0.10		
**Prior psychiatric treatment (yes)**	−1.33 (−2.60 to −0.07)	**0.04**	−0.79 (−2.23 to 0.66)	0.28	−0.48 (−1.98 to 1.01)	0.53		

Bootstrapped univariate mixed linear regression models were performed with the variable of interest as the independent variable and a random intercept for each participant. Each variable with a *p*-value of <0.10 in the univariate model was included in the multivariate model. Variables with a *p*-value < 0.5 in the multivariate model were identified as factors significantly associated with the outcome (highlighted in bold).

**Table 4:** Potential factors associated with mental health and physical health assessed by SF-12 among 95 admitted COVID-19 patients during May 2020–May 2021.

	SF-12 Mental Health				SF-12 Physical Health			
	Univariate		Multivariate		Univariate		Multivariate	
	Beta (95% CI)	*p*-Value	Beta (95% CI)	*p*-Value	Beta (95% CI)	*p*-Value	Beta (95% CI)	*p*-Value
**Time since discharge**								
1 month	1.06 (−1.43 to 3.55)	0.41	−1.50 (−3.76 to 0.77)	0.20	1.21 (−1.66 to 4.08)	0.41	0.17 (−2.38 to 2.73)	0.89
3 months	3.47 (0.30 to6.64)	**0.03**	−0.06 (−2.32 to 2.20)	0.96	4.24 (1.31 to7.16)	**<0.01**	2.19 (−0.14 to 4.52)	0.07
**Age, years**	0.01 (−0.07 to 0.10)	0.75			0.03 (−0.06 to 0.13)	0.53		
**Sex (female)**	−1.97 (−5.14 to 1.20)	0.22			−4.46 (−8.04 to −0.88)	**0.02**	−3.50 (−6.28 to −0.72)	**0.01**
**County of birth outside DK**	−2.88 (−6.41 to 0.65)	0.11			−3.19 (−6.95 to 0.60)	0.10		
**Education**								
Compulsory (middle/high school)	ref				ref			
Higher education (College/University)	1.23 (−1.74 to 4.20)	0.42			0.96 (−2.26 to 4.19)	0.56		
**Charlson’s Comorbidity**								
0	ref		ref		ref		ref	
1	−3.32 (−6.78 to 0.14)	0.06	−1.20 (−3.47 to 1.07)	0.30	−5.24 (−8.74 to −1.73)	**<0.01**	−2.98 (−5.68 to −0.29)	**0.03**
2	−5.54 (−9.11 to −1.96)	**<0.01**	−4.77 (−7.14 to −2.39)	**<0.001**	−6.03 (−9.83 to −2.22)	**<0.01**	−4.69 (−7.94 to −1.45)	**<0.01**
**Hospital stay, days**	0.02 (−0.18 to 0.22)	0.84			0.02 (−0.18 to 0.22)	0.82		
**ICU admission/mechanical intervention**	−2.88 (−20.96 to 15.21)	0.76			−6.42 (−22.38 to 9.55)	0.43		
**Prior psychiatric treatment (yes)**	2.05 (−1.11 to 5.21)	0.20			3.12 (−0.50 to 6.75)	0.09		
**Severity of anxiety symptoms**	−1.33 (−1.73 to −0.93)	**<0.001**	−0.14 (−0.60 to 0.33)	0.56	−1.23 (−1.66 to −0.79)	**<0.001**	0.03 (−0.40 to 0.47)	0.87
**Severity of depressive symptoms**	−1.34 (−1.74 to −0.94)	**<0.001**	−0.77 (−1.16 to −0.37)	**<0.001**	−1.25 (−1.72 to −0.78)	**<0.001**	−0.58 (−0.97 to −0.18)	**<0.01**
**Severity of perceived Stress**	−0.92 (−1.12 to −0.67)	**<0.001**	−0.43 (−0.68 to −0.19)	**<0.01**	−0.85 (−1.07 to −0.63)	**<0.001**	−0.50 (−0.79 to −0.22)	**<0.01**
**Severity of insomnia**	−0.82 (−1.09 to −0.54)	**<0.001**	−0.17 (−0.43 to 0.09)	0.20	−0.86 (−1.08 to −0.64)	**<0.001**	−0.32 (−0.57 to −0.07)	**0.01**
**Severity of PTSD symptoms**	−12.71 (−16.40 to −9.01)	**<0.001**	−3.10 (−8.03 to 1.83)	0.22	−12.25 (−15.23 to −9.26)	**<0.001**	−2.03 (−6.04 to 1.97)	0.32

Bootstrapped univariate mixed linear regression models were performed with the variable of interest as the independent variable and a random intercept for each participant. Each variable with a *p*-value of <0.10 in the univariate model was included in the multivariate model. Variables with a *p*-value < 0.5 in the multivariate model were identified as the factors significantly associated with the outcome (highlighted in bold).

## Data Availability

The data presented in this study are available upon reasonable request from the corresponding author and with approval from the Data Protection Agency. The data are not publicly available, as restrictions apply to the availability of these data, which were used under license for the current study.
